# Personality Traits and Psychotic Proneness Among Chronic Synthetic Cannabinoid Users

**DOI:** 10.3389/fpsyt.2020.00355

**Published:** 2020-05-15

**Authors:** Koby Cohen, Shiri Rosenzweig, Paola Rosca, Albert Pinhasov, Abraham Weizman, Aviv Weinstein

**Affiliations:** ^1^Department of Behavioral Science, Ariel University, Ariel, Israel; ^2^Ministry of Health (Israel), Jerusalem, Israel; ^3^Adelson School of Medicine, Ariel University, Ariel, Israel; ^4^Department of Molecular Biology, Ariel University, Ariel, Israel; ^5^Geha Mental Health Center, Petach Tikva, Israel

**Keywords:** synthetic cannabinoids, personality, psychosis, cannabis, addiction

## Abstract

**Objective:**

Chronic use of synthetic cannabinoids (SCs) has been associated with a wide range of negative consequences for health including psychotic and affective disturbances. Accumulating evidence indicates that cannabinoids use may be a risk factor for schizophrenia, and chronic natural cannabis users score higher than non-users on measures of schizotypal personality traits. However, little is known regarding the personality characteristics of SC users, especially in comparison with recreational cannabis users and healthy individuals. This study aimed to examine the differences in personality characteristics and schizotypy between SC users, regular cannabis users, and non-users and to compare these measures between groups.

**Methods:**

Forty-two chronic SC users, 39 natural cannabis users, and 47 non-using control participants, without history of mental disorder, or current substance use diagnosis (mean age 26± 4.47 years; 23 females, 105 males), completed the Big-Five Factor Inventory (BFI), the Schizotypal Personality Questionnaire-Brief (SPQ-B), substance use history, rating scales of depression and anxiety, and a demographic questionnaire.

**Results:**

On the BFI, SC users scored higher than natural cannabis users and non-users on neuroticism, but lower on agreeableness and extraversion, and endorsed greater schizotypal symptoms on the SPQ-B. In addition, SC users had lower scores on conscientiousness than non-users, and natural cannabis users were more extroverted than non-users. Higher openness and lower conscientiousness predicted schizotypy for both SC and natural cannabis users. Finally, greater neuroticism predicted schizotypy for natural cannabis users, and introversion predicted schizotypy for non-users.

**Conclusions:**

These results show that chronic SC users differ from natural cannabis users and non-users on dimensions of specific personality traits and schizotypy that may indicate psychotic proneness.

## Introduction

### Epidemiology

Cannabis is the most popular recreational psychoactive substance following tobacco and alcohol (1). Around 4% of the global adult population has used cannabis in their life. In the United States of America (USA) alone, at least 36 million people used cannabis at least once in their lifetime (1, 2). Since several countries have conducted a decriminalization policy regarding the possession of cannabis for recreational use and possession of cannabis in small amounts (1, 3), it seems likely that the consumption of cannabis will increase in the coming years (4). Recently, a new type of cannabinoid-based drugs has started to be consumed recreationally among drug users across the globe (5, 6). These new cannabinoid-based drugs classified as novel psychoactive substances (NPS) and are composed a high concentration of SCs (7–10). Drug brands such as “Spice” and/or K2 are generally used to describe the diverse types of herbal blends that encompass synthetic cannabinoids (SCs), same as other NPS, individuals who consume SCs are typically attracted by these substances due to their intense psychoactive effects and likely lack of detection in routine drug screenings (10–12). Lifetime prevalence of SC use in the general population is similar to other NPS and ranges between 0.2 to 4% (13). Contrary to other types of NPS, SC use has not been associated with low educational levels or low incomes (14) and SC users are mostly young males, high school graduates using other recreational drugs (15). Since the beginning the current decade, the existence of more than a hundred different types of SCs were documented by the European Union Early Warning System. These drugs are mainly sold online as a “legal” alternative to controlled and regulated psychoactive substances. They appear to have a life cycle of about few years before being replaced by a next generation of products. Regulation controlling these NPS has been introduced in several states in order to limit the spread of existing drugs and control potential new analogs (16).

### Neurobiology

Synthetic cannabinoids compared with natural cannabis have higher affinity with endogenous cannabinoid receptor type-1 (CB_1_) and/or endogenous cannabinoid receptor type-2 (CB_2_) with a high affinity/potency and they are full receptor agonists [Δ9-tetrahydrocannabinol (THC) is a partial agonist]. Unlike natural cannabis, in SCs there is no cannabidiol (CBD) (which may protect against psychosis) and they have longer half-life active metabolites. Their; effects are more intense and longer lasting, bringing greater health risks, more powerful, and unpredictable effects, with higher toxicity and overdose potential than THC. SCs are mainly consumed by smoking; solely or with cannabis, when absorbed, SC induce a wide range of adverse effects, some of them are similar to the psychotropic effects of cannabis (7–9). However, the acute effects are more intense, in terms of duration and severity induce both somatic and psychiatric adverse effects (8, 9, 12, 15, 17, 18). Although the chronic toxicity of SC is still not well known, recent studies have found neuronal alterations, cognitive impairments, and mental distress in chronic SC users (19–22). Interestingly, recent data has indicated that SC hold a greater risk for psychosis compared to regular (non-synthetic) cannabis (23, 24). Previous studies have linked chronic use of cannabis to personality dimensions associated with increased psychosis-proneness, or schizotypy (25–27). However, there is limited information regarding the personality characteristics of SC users and the relations of personality and schizotypy to this population. The identification of personality traits specific for SC users could be useful for the development of effective screening instruments and future prevention and intervention strategies of psychosis-proneness in this population (28–30). The main object of the current study was to explore the personality characteristics of SC users compared with those of cannabis users and non-user subjects, and to examine the relationships between personality factors underline schizotypy in SC users compared with regular cannabis users and non-users.

### Synthetic Cannabinoids and Related Adverse Effects

Similar to regular cannabis, the primary psychoactive constituents of SC drugs interact with CB_1_ and CB_2_ receptors (5, 7, 9, 31, 32). There is an agreement that the activation of CB_1_ receptors following consumption of an exogenous cannabinoid-agonist may underline the psychoactive effect of cannabinoid-based drugs (33–36). In contrast to regular cannabis, SCs contain extremely potent CB_1_-receptor full-agonist as well as additional psychoactive ligands, and are missing anti-psychotic CB_1_-receptor-antagonist ligands such as CBD (5, 7, 9, 31, 32, 36). Furthermore, SC drugs are composed of variable concentrations of a wide range of other ingredients, have a longer half-life active metabolite, and induce long-lasting and unpredictable adverse effects bringing greater health risks with higher toxicity and overdose potential than regular cannabis (23, 32, 36). These features hold by SC drugs may indicate their great harmful-potential (37, 38).

Although the acute and chronic toxicity related to SC use is still not well known, the negative consequences associated with SCs include a variety of psychoactive effects, such as: mood alterations, anxiety, paranoia, cognitive impairment, dissociation, excitability and agitation, sedation, and psychosis (17, 24, 37, 39–42). The use of SC may trigger the occurrence of severe psychosis in psychosis-prone users or the exacerbation of a prodromal psychotic syndrome in healthy individuals, due the rigid psychopathological issues associated with SC intoxication it is sometimes referred to as “spiceophrenia” (18). In addition, physical effects included nausea, vomiting, diarrhea, tremors, hypertension, tachycardia, and symptoms of dependency (24, 37, 39–42) **Table 1** describes clinical side-effects of synthetic cannabinoids.

**Table 1:** Summary of clinical side-effects of synthetic cannabinoids.

Acute psychopathology	Agitation, manic episode, anxiety, irritability, disorganized behavior, violent behavior, aggression, altered visual/auditory perception or hallucinations, delusion, confusion, altered attention and concentration, amnesia and memory impairment, paranoia, mood alterations, suicidal ideation, sedation, catatonia, (6, 7, 12, 17, 24, 37–50). Chronic use may increase the risk for developing psychotic disorders (18, 32, 36, 46).
Other acute toxicity	Tachycardia, drowsiness/lethargy, hypertension, headache, nausea/vomiting, tremor, dizziness/vertigo, ataxia, dysarthria, angina, palpitations, dyspnea, mydriasis, bradycardia, hypotension, rhabdomyolysis, seizures, stroke, arrhythmias, myocardial infarction, emboli, encephalopathy, acute kidney injury, coma, including agitation, mydriasis, diaphoresis, tremor, clonus, hyperreflexia, hyperthermia (38, 42–45).
Possible long-term adverse effect	Adverse effect on cognitive functions including; memory alteration, attention difficulties, thinking problems, and slow responses, alterations in brain’s structure and functions (20–22, 51, 52). Chronic use may increase the risk for developing psychopathology including mood and psychotic disorders (38, 42–45).

Some symptoms such as cardiovascular events, seizures, agitation, hypertension, emesis, and hypokalemia are features of SC intoxication and are not present even after consuming high doses of regular cannabis (19, 24). Severe toxicity due to SCs has been required medical intervention mostly of neuropsychiatric and cardiovascular clinical manifestations (43–45). Since the use of SC is rapidly growing together with it increasing health related events recent works have suggested that actions such as; prompt reliable information available for health professionals, more specific analytic techniques, designed preventive strategies for at-risks categories, and for law enforcement strategy in the commune are all required to face the SC phenomena (53).

Recently, several cohort studies have shown evidence for cognitive deficits and affective alterations in chronic SC users (20–22, 46). Complementary neurobiological studies demonstrated in chronic SC users alterations in brain regions which are involved in cognitive and emotional function (22, 51, 52). The evidence of neuronal damage associated with chronic use of SC is alarming, since it may indicate possible neurotoxic effects of SC drugs (22, 51, 52). Moreover, SC use is common among teenagers and young adults, who are more vulnerable to the negative impact of cannabinoids on the central nervous system (CNS) (19, 54–56).

### Cannabinoids and Personality Factors

Beside early age, other factors such as personality predisposition are associated with cannabis use and are linked with greater vulnerability to the adverse effect of cannabinoid use (25–27, 57). Personality characteristics such as sensation seeking, anxiety, and emotional distress are associated with early onset of drug use (29, 58), and greater levels of emotional imbalance and extraversion are associated with increased risk for developing psychosis following chronic regular cannabis use (25). A large-number of studies describes the association between drug-abuse and personality characteristics. Although several studies examined the motives and demographic characteristic of SC users, yet, there is no available information on the personality dimensions for this population (11, 47, 59, 60). On the other hand, there are few studies that have characterized the personalities of chronic regular cannabis users. Flory et al. (61) have found that symptoms of cannabis dependency were negatively correlated with agreeableness and conscientiousness and were associated with openness. However, after controlling additional factors such as alcohol consumption, antisocial personality disorder, and internalizing disorders, cannabis dependency was positively correlated with openness and negatively associated to extraversion (61). Later on, Terracciano et al. have conducted an epidemiological study and found that current cannabis users were lower in agreeableness and conscientiousness, but higher in openness, relative to healthy non-users control subjects. However, in their study additional confounding factors were not controlled (62). Allen and Holder have found that regular cannabis use has been associated with lower agreeableness and lower conscientiousness (63). Similar result was found by Tartagila et al. who showed an association between cannabis use and low levels of agreeableness and conscientiousness, and higher levels of openness in a sample of university students (64). In an epidemiological study conducted by Hengartnet et al. cannabis consumption has been found to be associated with higher scores on extraversion and openness, and lower scores on conscientiousness (28). Altogether, these studies provide strong evidence on the association between personality traits and regular cannabis use, yet the pattern of the result is inconsistent, possibly due to the heterogeneity in the studied populations and confounding factors. Interestingly, Friedberg and colleagues have shown schizotypal features that were common among cannabis users and had been associated with certain personality characteristics. In their study, they have compared a group of cannabis users with a group of healthy drug-naïve control subjects and they have found higher scores of openness, and lower levels of agreeableness and conscientiousness in regular cannabis users compared with control subjects. Moreover, higher levels of neuroticism predicted schizotypy in all participants and extraversion predicated negative schizotypal symptoms (25). Greater levels on schizotypal measures were reported in earlier several studies in regular heavy cannabis users (25–27, 65–67). However, it is unclear whether the association between schizotypal symptoms and regular cannabis use is a result of repeated cannabis use, inherited predisposition, or additional confounding factors (25, 67).

### Rational and Aims of the Current Study

The main aim of the present study was to investigate the personality characteristics of SC users compared with natural cannabis users and non-users on measures of the Big-Five Factors (BFI) (68, 69) and schizotypy (70), and to examine the relations among those measures within each group. To our knowledge, this is the first study to investigate the personality profile of SC users compared with natural cannabis users. We hypothesized that SC users would show higher levels of schizotypy compared with natural cannabis users and non-users, suggesting greater psychosis-proneness. In addition, we have predicted that SC users would present greater levels of neuroticism and introversion and lower conscientiousness, than the two control groups. Finally, we plan to investigate the contribution of depression, anxiety, and personality traits to the variance of schizotypal scores in all groups.

## Methods

### Participants

One hundred and twenty-eight participants were recruited for the study, including 105 males and 23 females. The mean age was 26.21 (SD=4.46) years. The total sample was divided to three groups based on their self-reported substance use history: a) SC users, b) regular cannabis users, and c) non-users. SC users were recruited from the Israeli Ministry of Health drug addiction treatment programs. Both regular cannabis users and non-users were recruited by convenient sampling *via* friends, relatives, or social networks.

#### Synthetic Cannabinoid Users

The SC users group comprised of 42 subjects, 32 males, and 7 females, who have frequently consumed SC drugs during the last 2 years. We have defined the inclusion criteria for SC users as a regular use on a monthly basis with a minimal usage of at least 10 times in the last year and without binge consumption defined as more than 4 usages of SC during the last month. The mean age was 27.1 (SD=5) years. Participants were cannabinoid-free for at least 1 week prior the study, were evaluated by a senior psychiatrist and diagnosed as not suffering from current psychosis or comorbid psychiatric or neurological disorders or a past or current substance use disorder other than cannabinoids.

#### Natural Cannabis Users

The group of natural cannabis users included 32 males and 7 females. Altogether, there were 39 subjects that used cannabis on a monthly basis with minimal usage of at least 10 times in the last year and without binge consumption defined as more than four usages of cannabis during the last month and they were cannabinoid-free for at least 1 week. The mean age in the natural cannabis user group was 25.25 (SD=3.51) years. Exclusion criteria for natural cannabis participants were history of neurological or psychiatric disorders and history or current or past substance use disorder other than cannabis.

#### Non-Users

The group of non-users included 40 males and 7 females, altogether 47 healthy individuals, who have reported that they did not consume cannabinoid-based drugs during the last 2 years. The participants’ mean age was 26.2 (SD=4.5) years. Exclusion criteria for healthy control participants were history of neurological or psychiatric disorders and history or current substance use disorder.

### Ethical Approvals

The Ariel University Review Board and the Israeli Ministry of Health have approved the study. All participants volunteered to participate in the study and did not get any reward for their participation. All the participants were above the age of 18 years, and signed an informed consent prior to participation.

### Materials and Design

#### Sample Characteristic and Substance Use History

The demographic questionnaires included items on education level, age, gender, and information regarding current or past neurological or psychiatric disorders. The questionnaires also contained items regarding the use of psychoactive substances, focusing on cannabis and SCs, as well as tobacco and alcohol. Data on the age of first use, the frequency of usages past month, and past year of cannabis and SC use were recorded.

#### Depression and Anxiety Levels

Depression and anxiety symptoms levels were recorded as well using the Beck Depression Inventory (BDI) (Cronbach internal reliability of α = 0.91) (71, 72), and the Spielberger State-Trait Anxiety Inventory (STAI-S, STAI-T) (Cronbach’s α = 0.91 and 0.85; respectively) (73).

#### Big-Five Factors Inventory

The BFI questionnaire was used to asses personality traits (68, 69). The BFI consists of 44 self-rated items on a five severity scores from 1 =strongly disagree to 5 =strongly agree. Each item represents one of the core traits that define each big five domains; extraversion, neuroticism, agreeableness, conscientiousness, and openness to experience. Total mean scores for each of the personality factors were recorded for each participant. The Hebrew version of the BFI was translated and validated previously, Cronbach’s α reliability of the Hebrew version domains ranged from 0.63 to 0.83 (74). In this study, the BFI Cronbach’s α reliability score ranged from 0.86 to 0.34.

#### Schizotypal Personality Questionnaire

The Schizotypal Personality Questionnaire-Brief (SPQ-B) was used to measure psychotic proneness (70). The SPQ-B is a 22-item (true/false) self-report for the assessment of schizotypal personality disorder or dimensional schizotypy. The SPQ-B consists of three subscales: a) cognitive-perceptual deficits, b) interpersonal problems, and c) disorganized symptoms. Each “true” response counts as one-point, total scores ranging from 0 to 22. The internal consistency indices of the SPQ-B ranged from 0.75 to 0.83 (from 0.58 to 0.83 for the subscales) and the test–retest reliability from 0.82 to 0.90 (75). In this study, the SPQ-B had a Cronbach internal reliability of α = 0.87.

### Statistical Analysis

The analysis of the results was performed on Statistical Package for Social Science (SPSS) for windows v.21 (IBM Corp. Armonk, NY). Differences between groups in terms of gender were tested using chi-square test and a multivariate analysis of variance (MANOVA) was used to calculate the effect of group on BFI domains, further one-way ANOVAs indicated the sources of significant group main effects. One-way ANOVA was conducted to examine group main effects on SPQ-B overall and sub-scale scores; Student’s t-tests followed by Bonferroni *post hoc* corrections were used for group comparisons. In additional analyses, anxiety rates and depression were added as covariate factors to the initial models in order to explore the possibility of confounding variables. Finally, hierarchal regression models were computed separately for each group in order to explore relationships between SPQ-B and BFI factors, depression and anxiety.

## Results

### Sample Characteristics and Substance Use History

Participant’s drug use history and demographic data are described in **Table 2**. Groups did not differ by gender, age, education level, or by rates of alcohol use history. SC users have consumed more tobacco cigarettes per day than either non-users and natural cannabis users SC users had used cannabinoid-based drugs at an early age than natural cannabis users In addition, SC users have scored higher on the BDI than non-users and natural cannabis users but there were no differences in BDI scores between natural cannabis and non-user groups SC users had higher scores on STAI Trait and State scales compared to natural cannabis users and non-users. There were no differences in STAI State and Trait scores between natural cannabis users and non-users.

**Table 2:** Demographic and questionnaires’ ratings in all participants.

	Synthetic	Cannabis	None	Comparison	Significance
N, frequencies (male: female)	42 (32:7)	39 (32:7)	47 (40:7)	^a^ 0.63	*p* > 0.05
Age, mean (SD)	27.1 (5)	25.25 (3.51)	26.2 (4.5)	^b^1.63	*p* = 0.2
Education level (SD)	12.3 (2)	12.1 (0.9)	12.8 (2.17)	^b^1.5	*p* = 0.21
Alcohol consumption (SD)	2.83 (2.61)	3.74 (1.72)	3.54 (2.55)	^b^1.99	*p* = 0.14
Tabaco consumption (SD)	16.85 (10)	4.82 (6.6)	2.7 (4.66)	^b^43.78	*p* < 0.001
Age of first use for cannabinoids	15 (6.56)	18.41 (4.72)	–	^c^2.66	*p* < 0.01
Frequency of cannabinoids use during the last month	21.53 (93.7)	19.5 (32.82)	–	^c^0.13	*p* = 0.89
Frequency of cannabinoids use during the last year	208 (146.31)	185 (134)	–	^c^0.75	*p* = 0.45
BDI, mean (SD)	39.19 (8.7)	27 (7.73)	24.9 (4.37)	^b^49.4	*^d^p* < 0.001
STAI Trait, mean (SD)	49.64 (6.58)	34.35 (7.47)	34.84 (8)	^b^51.54	*^d^p* < 0.001
STAI State, mean (SD)	48.38 (7.4)	32.97 (9.91)	32.84 (9.54)	^b^41.13	*^d^p* < 0.001

Age and education level reported in years; alcohol consumption habits drink defined as glass of wine or 250 ml of beer or one shot of alcoholic beverages; tobacco consumption, cigarettes per day; frequencies of cannabinoids use defined as event of consummation of cannabinoid-based drugs; BDI, Beck Depression Inventory scores; STAI, Silberberg Trait or State Anxiety inventory scores; SPQ-B, Schizotypal Personality Questionnaire Brief; significant level of difference between drug groups within the total sample; ^a^χ^2^; ^b^F(2,125); ^c^t(78), differences observed for SC vs. cannabis; ^d^t(78), differences between SC vs. cannabis users, and t(84) SC vs. non-users.

### The Big-Five Factors

**Figure 1:**
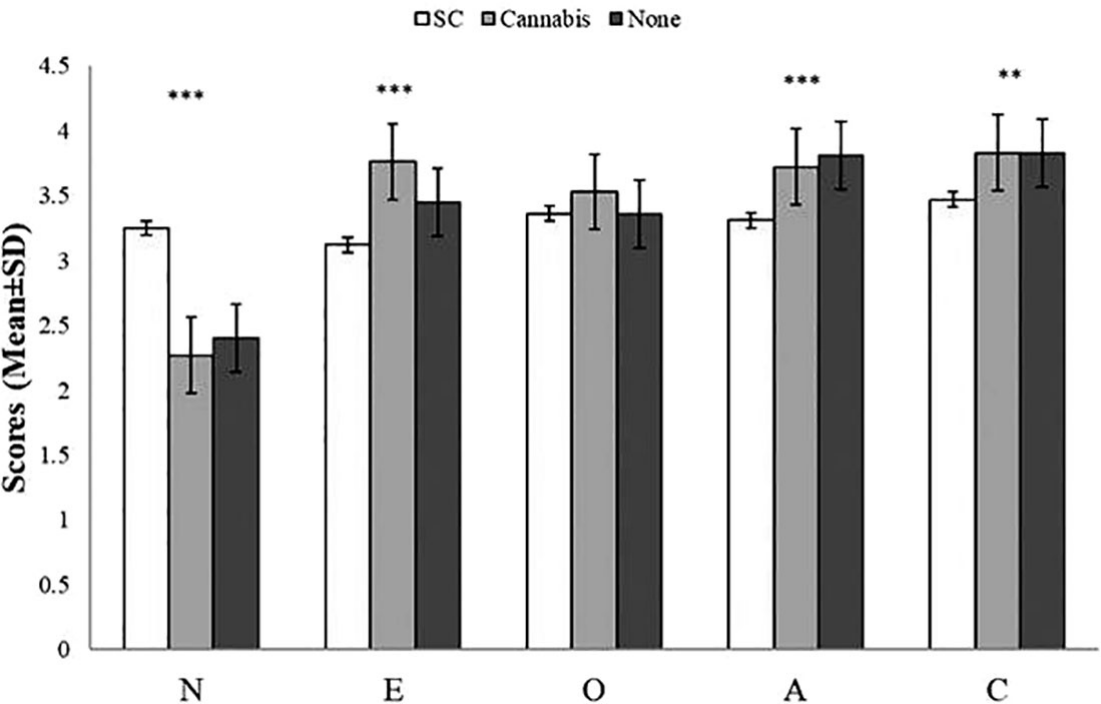
Scores (mean ± SD) of the Big-Five Factor Inventory (BFI) sub-scales by group. There was a main effect of group on neuroticism (SC>Cannabis, SC>Non-users, Cannabis=Non-users), extraversion (SC<Cannabis, SC<Non-users, Cannabis>Non-users), aggreableness (SC<Non-users, SC<Cannabis, Cannabis=Non-users), and conscientiousness (SC<Non-users, SC=Cannabis, Cannabis=Non-users); there were no differences among group in openness; ***p < 0.0001, **p < 0.01; A, agreeableness; C, conscientiousness; E, extraversion; O, openness; N, neuroticism.

The mean scores on BFI factors by group are presented in **Table 3**. Initial analysis showed a significant effect of the groups on BFI [Wilks’ lambda=0.61, *F* (10, 240) = 6.91, *p* < 0.001]. Further one-way ANOVAs have indicated a main effect of group on neuroticism, extraversion, conscientiousness, and agreeableness. SC users had higher neurotic scores, lower ratings of agreeableness, and lower ratings of extraversion, than natural cannabis users and non-users. Furthermore, SC users had lower conscientiousness scores than non-users yet, there were no differences in conscientiousness between SC and natural cannabis users and between natural cannabis users and non-users. The groups did not differ on scores of openness (**Figure 1**). Finally, when anxiety or depression were entered to the MANOVA as covariate factors, the effects of group on neuroticism [*F*(2,121)= 1.31, *p*=0.27; *F*(2,121)= 4.07, *p* < 0.05, respectively], extraversion [*F*(2, 121)= 4.49, *p* < 0.05; *F*(2,121)= 5.48, *p* < 0.01, respectively], conscientiousness [*F*(2, 121)= 1.27, *p*=0.28; *F*(2,121)= 1.51, *p*=0.22, respectively], and agreeableness [*F*(2, 121)= 3.05, *p*=0.05; *F*(2,121)= 2.7, *p*=0.07, respectively] were reduced.

**Table 3:** Mean scores of groups for each of the Big-Five Factor Inventory (BFI) sub-scales.

	Group			Comparison	
	SC	Cannabis	None	*F*(2,125)	*P*-value
Extraversion	3.12 (0.51)	3.76 (0.74)	3.45 (0.55)	12.26	*^a^^b^p* < 0.001
Neuroticism	3.25 (0.63)	2.27 (0.69)	2.4 (0.76)	24.10	*^a^p* < 0.001
Agreeableness	3.31 (0.51)	3.72 (0.61)	3.81 (0.55)	8.45	*^a^p* < 0.001
Conscientiousness	3.47 (0.52)	3.83 (0.44)	3.83 (0.44)	5	*^c^p* < 0.01
Openness	3.35 (0.65)	3.53 (0.47)	3.36 (0.47)	0.9	0.42

Values are mean (SD).^a^Difference observed for SC vs. cannabis and for SC vs. non-users; ^b^differences observed for non-users vs. cannabis; ^c^difference observed for SC vs. non-users.

### Schizotypal Personality Questionnaire

**Table 4** shows Schizotypy questionnaire dimensions by all groups of participants.

**Table 4:** Schizotypy questionnaire dimensions by groups.

	Synthetic	Cannabis	None	Comparison	
				*F*(2,122)	*p-*value^a^
SPQ-B total score	11.64 (5)	5.35 (3.58)	4.22 (3.19)	41.98	*p < 0.001*
SPQ-B cognitive-perceptual	4.57 (2.27)	1.76 (1.5)	1.43 (1.46)	38.73	*p < 0.001*
SPQ-B interpersonal	3.88 (2.26)	2.10 (1.97)	1.84 (1.52)	13.84	*p < 0.001*
SPQ-B disorganized	3.19 (1.75)	1.48 (1.51)	0.95 (1.18)	25.77	*p < 0.001*

a Difference observed for SC vs. cannabis and for SC vs. non-users.

Analysis revealed the main effect of group on SPQ-B scores. SC users had greater score on the SPQ-B compared with natural cannabis users [*t* (79) = 6.44, *p* < 0.01] and non-users [*t* (87) = 6.84, *p* < 0.01]. There were no differences on the SPQ-B between natural cannabis and non-users [*t* (84) = 0.47, *p*=1.00]. There were main effects of groups on SPQ-B’s sub-scales: cognitive-perceptual, interpersonal, and disorganization. SC users have scored higher than natural cannabis users [*t*(79)= 2.80, *p* < 0.001 and *t*(79)=3.76, *p* < 0.001; *t*(79)= 4.65, *p* < 0.001, respectively] and non-users [*t*(87)= 6.95, *p* < 0.001, *t*(87)= 4.20, *p* < 0.001 and *t*(87)= 5.53, *p* < 0.001, respectively]. There were no differences between natural cannabis users and non-users in either cognitive-perceptual [*t* (84) = 0.70, *p*=1.00], interpersonal [*t* (84) =0.04, *p*=1.00], or disorganization [*t* (84) = 0.75, *p*=1.00] sub-scales. The effect of group on SPQ-B score remained significant when anxiety [*F*(2,121)=17.87, *p* < 0.001], and depression [*F*(2,121)=16.37, *p* < 0.001] were used as covariates, a similar pattern was observed for SPQ-B’s sub-scales; cognitive-perceptual [*F*(2,121)= 20.54, *p* < 0.001; *F*(2,121)= 18.04, *p* < 0.001, respectively], interpersonal [*F*(2,121)= 3.26, *p*= < 0.05; *F*(2,121)=3.12, *p* < 0.05, respectively], and disorganization [*F*(2,121)= 12.61, *p* < 0.001; *F*(2,121)= 11.68, *p* < 0.001, respectively].

### Association Between Schizotypal and Personality

In order to explore the relationships between schizotypal trait and personality factors a serial of hierarchical multiple regression analyses was conducted; first for the whole sample and then for each group separately with the schizotypal scores as a dependent variable and BFI domains as predictors. In order to account for group differences, depression and anxiety variables were entered in the first step of the model and scores of all BFI factors were entered in the second step. In the first repression model, personality traits significantly contributed to the variance of schizotypy after controlling for depression and trait and state anxiety scores. Beside depression, higher scores of openness and neuroticism and lower scores of extraversion predicted schizotypy. Specific analysis of each group showed that high scores of openness and lower scores of conscientiousness predicted schizotypy for SC and natural cannabis users. Finally, greater neuroticism predicted schizotypy for natural cannabis users and introversion predicted schizotypy for non-users. **Table 5** shows hierarchical multiple regression analysis predicting schizotypal scores by the scores of depression, anxiety, and personality traits for the three groups.

**Table 5:** Hierarchical multiple regression analysis predicting schizotypal scores by the scores of depression, anxiety, and personality traits for the three groups.

	Factors	B	β	Total R^2^	*F* Value	*p-*Value
Total sample	First step			0.31	26.57	*p* < 0.0001
	Depression	0.22	0.41** ^II^			
	Anxiety	0.08	0.17			
	Second step			0.46	14.06	*p* < 0.0001
	Neuroticism	2.15	0.34**			
	Extraversion	−1.67	−0.22**			
	Openness	1.47	0.19*			
	Conscientiousness	−1.4	−0.15^¥^			
	Agreeableness	−0.1	−0.01			
Non-users	First step			0.16	3.84	0.03
	Depression	0.22	0.3			
	Anxiety	0.05	0.11			
	Second step			0.43	3.92	0.003
	Neuroticism	1.31	0.28			
	Extraversion	−2.6	−0.46**			
	Openness	−0.4	−0.06			
	Conscientiousness	0.93	0.12			
	Agreeableness	0.65	0.12			
Cannabis	First step			0.09	1.72	0.19
	Depression	0.12	0.23			
	Anxiety	0.04	0.1			
	Second step			0.47	3.78	0.005
	Neuroticism	2.74	0.52*			
	Extraversion	−0.92	−0.20			
	Openness	2.55	0.36*			
	Conscientiousness	−1.79	−0.34*			
	Agreeableness	−0.52	−0.9			
Synthetic	First step			0.12	2.53	0.09
	Depression	0.13	0.25			
	Anxiety ^II^	−0.31	−0.41*			
	Second step			0.43	3.53	0.006
	Neuroticism	1	0.13			
	Extraversion	−1.31	−0.45			
	Openness	2.02	0.39*			
	Conscientiousness	−4.16	−0.46 *			
	Agreeableness	0.57	0.08			

Depression and anxiety scores were entered simultaneously in step 1; rhe scores of depression (Beck Depression Inventory), anxiety (mean score of both Spielberger state and trait anxiety inventory), and Big-Five Factor Inventory (BFI) personality traits were entered simultaneously in step 2;*p < 0.05, **p < 0.01, ^II^p < 0.01 in step 2, ^¥^p = 0.05.

## Discussion

The purpose of the present study was to explore the personality characteristics of SC users and compare them to those of natural cannabis users and non-users. Our results showed that chronic SC users differ from both natural cannabis users and non-users on the BFI personality traits and schizotypy measures. On the BFI, SC users had higher scores of neuroticism and lower scores of agreeableness and extraversion compared with natural cannabis users and non-users. In addition, SC users have presented lower levels of conscientiousness relative to non-users, and similar scores of openness compared to both control groups. These results are consistent with previous studies that showed an association between drug use disorders including cannabis and higher neuroticism, lower conscientiousness and agreeableness, and scores on the extroversion-introversion scale (29, 62, 76–82).

### Synthetic Cannabinoids and Neuroticism

Neurotic individuals usually experience high levels of negative affect, suffer from anxiety and depression, and have a low activation threshold in the face of external or internal stressors (83). SC users in this study, as in previous studies, also showed elevated symptoms of depression and anxiety (21, 22, 84). According to recent studies on the role of neuroticism in the etiology of addictive disorders, high levels of neuroticism predispose individuals to both personality and substance use disorders. Thus, neurotic individuals are prone to use psychoactive agents which accord their excessive physiological arousal (85). Interestingly, neuroticism was found to be associated with cocaine, opioids, and amphetamine use (69, 79, 86). However, the association between neuroticism and cannabis is mixed, as it seems to be influenced by additional factors such as: extensive cannabis use, mood, anxiety, and psychiatric conditions (25, 64, 87). Chowdhury et al. (2015) have found an association between cannabis and neuroticism among regular cannabis users in a community-based study. Yet, most of the participants in their research have been met the criteria of depressive disorder, generalized anxiety disorder, or alcohol abuse (87). In addition, the sample contained a mixture of both regular and occasional cannabis users. Later-on, a series of studies have failed to show an association between neuroticism and cannabis use, these investigations composed samples of cannabis user who did not suffer from psychiatric symptoms as well as current substance-abuse (25, 63, 64). Furthermore, similar to the present study, the samples of cannabis users in these studies (25, 63, 64) were composed from recreational cannabis users. Thus, it is reasonable to assume that regular cannabis users show high neuroticism whereas recreational users show low neuroticism. The low neuroticism score in recreational cannabis users in our study is therefore since our subjects were not regular users and did not show the psychological distress that may affect the association between cannabis use and neuroticism (25, 63, 64).

### Synthetic Cannabinoids and Conscientiousness

SC had lower scores on conscientiousness compared to non-users as well as lower scores of agreeableness than both control groups. Recent reports had indicated that SC users were prone to manifest antisocial behaviors and tend to be aggressive, manipulate, impulsive, and hostile toward others (47, 84, 88, 89). Low levels of conscientiousness are often associated with impulsivity, mental distress, risk taking behaviors (including health risks), and maladaptive coping strategies (69). Low scores of conscientiousness not only enhance the chance of health risk taking behavior, but also affect the mechanisms which regulate the maintenance of drug abuse (90). Low scores of agreeableness are associated with emotional detachment from others, suspiciousness as well antagonism, and dishonesty (91). Together, low agreeableness and conscientiousness characterize the personality profile of chronic drug users (92, 93). Accordingly, low levels of these traits may predispose individuals to abuse substances or may account for problems in interpersonal relationships which are commonly associated with drug use disorders (94). The low levels of agreeableness and conscientiousness of SC users in the current study may be associated with difficulties with authority and health risk taking behaviors in SC users (29, 94, 95).

### Synthetic Cannabinoids and Extraversion

SC users also showed lower scores on extraversion (i.e., *introverts*), while natural cannabis users had higher scores on extraversion (i.e., *extroverts*) compared with non-users. Introverts are less interested in the external world, they are imaginative, tend to live within themselves, and they avoid referring themselves to social supports in order to minimize confrontation with stressful situations (96). Higher scores on this scale are associated with greater risk for suicidal attempt (97). Previous studies have linked SC use with suicidal ideation and relatively high incidence of suicide attempts (98–100). On the other hand, extroverts tend to be sociable and sensation seeking, they often exhibit lack of behavioral constrain and fail to conform to conventional norms (100). Consistent with the former, Bozkurt and colleagues (2014) have found that SC users preferred to use SC alone rather than with a companion (59), and a recent report indicated that users attempted to consume SC secretly (60). Additionally, drug users prefer to communicate with others *via* social media rather than direct interpersonal communication (92). Contrary to SC users, natural cannabis use is common in social settings (101), and extroverts may appreciate and pursue the social ritual and support associated with cannabis use (102). The former indicates that while SCs are commonly used individually and secretly, regular cannabis is mostly consumed in a group setting, as a part of social activity. This observation is consistent with the differences between SC and natural cannabis users in extraversion levels obtained in our study. However, the association between cannabis use and extroversion is inconsistent. Flory et al. (61) have reported that introversion has been associated with cannabis dependence (61). On the other hand, Hengartnet et al. (2016) showed that extraversion is associated with cannabis use (28). Later on, several studies have reported no association between introversion or extraversion and natural cannabis users (25, 62–64). Importantly, while acute intoxication of cannabis has been associated with increased levels of sociability and empathy toward others (103), chronic natural cannabis use often induces “amotivational syndrome,” a psychological condition in which social withdrawal is considered to be a prominent expression (104, 105). It is possible that the high introversion scores in SC users may reflect this syndrome, as a result of chronic consumption of potent SCs. The natural cannabis users were young adults who smoked relatively small amounts of low-potency cannabis and therefore may not show the “amotivational syndrome” and this may explain the differences between SC and regular cannabis users, at least with regard to introversion. Finally, the current study suggests that neuroticism, low agreeableness, high introversion, and low conscientiousness are the personality characteristics of SC users. Although there is insufficient evidence on an exclusive personality profile for drug users (106), our results are in accord with a well-designed meta-analysis study which showed that a personality profile of high neuroticism, low agreeableness, introversion, and low conscientiousness is associated with a wide range of psychiatric disorders (107). Accordingly, a common pattern of personality characteristics is observed in drug dependent individuals regardless of the specific drug being used (107). In light of the current results, it is reasonable to assume that individuals with increased risk for developing drug use disorder tend to use SC drugs. Moreover, it may further imply that SC users and natural cannabis users represent a different type of population.

In summary, the personality characteristics that were identified in the current study for SC users are: a) consistent with previous studies described behavioral and psychological symptoms in chronic SC users, b) may underlie part of the psychological mechanisms of SC addiction, and c) may indicate that SCs attract individuals with a unique, problematic personality characteristics, which are different from natural cannabis users.

### Synthetic Cannabinoids and Psychosis

We have found that SC users have shown greater scores of schizotypy traits compared to natural cannabis users and non-users. This finding accords previous indications for the association between psychotic proneness and chronic cannabis use disorder (25–27, 65–67, 108). The association between cannabinoids and psychosis is well documented and recognized (1, 19, 33, 35, 54, 109–112). Converging data suggests that cannabis use has the potential for inducing psychosis (1, 19, 33, 35, 54, 109–112). The evidence may explain the relatively high incidence of severe psychosis that have been observed in chronic SC users. Vallersnes and colleagues (2016) have reported that SCs were the drugs most frequently involved in presentation of psychosis to an emergency department in Europe (48). In England, 28% of the SC users who were referred to health professionals due to SC intoxication had presented a severe psychotic episode (45). Recent reports in Europe suggest that 15% of SC users who report to emergency departments present psychotic symptoms (48). These figures are far greater compared to those using other types of psychoactive substance (48). In addition, compared with natural cannabis, psychotic symptoms that are associated with SC are more aggressive and rigid, accompanying with elevated and prolonged mental distress (49, 84, 89). In an Israeli retrospective cohort study, Shalit et al. retrieved data from a period of 7 years in order to examine demographic and clinical characteristic of SC users admitted to a mental-health center in comparison to regular cannabis users. Patients admitted following use of SC had higher severity of psychotic symptoms, were more likely to be admitted by criminal court order, and required longer hospitalization periods in comparison to regular cannabis users (84). However, SC users have not shown higher rates of depression, anxiety, or physiological symptoms compared with natural cannabis users. Recently, Mensen have investigated mental health consequences associated with SC use in a non-clinical sample (47). The authors have shown that compared to natural cannabis use, SC use is more strongly associated with a broad range of self-reported mental health problems such as: sleep problems, manic ideation, somatization, obsessive-compulsive behaviors, hyper interpersonal sensitivity, depression, anxiety, hostility, phobic anxiety, paranoid ideation, and psychoticism. They have suggested that there are more severe problems related to SC use compared to natural cannabis use (47). Consistently, recent studies have indicated that SC use is associated with greater psychoticism, and a broad range of psychological symptoms. There are differences between the population in Shalit’s study of patients that were admitted to hospital due to psychotic episodes and the population in our study who were not admitted due to psychosis and the general population reported by Mensen (47, 84). These differences may account for the variations in the adverse effects of SC drugs, including anxiety and depression.

### Possible Mechanisms for the Association Between Synthetic Cannabinoids and Psychosis

A possible explanation for SC induced psychosis is that SC products contain compounds which act as highly potent CB_1_ and CB_2_ full agonists, and in contrast to natural cannabis, contain no CBD (5, 7, 9, 31, 32, 36). Due to the psychoactive features of SC drug ingredients it is not surprising that there are numerous reports on healthy and vulnerable individuals who suffer from recurrent psychosis after an acute or repeated consumption of SC drugs (37, 38). Converging evidence suggests that the adverse effects of cannabinoids are dose-dependent, thus, as the concentration of the CB_1_ agonist increases, the adverse effects of cannabinoid-based drugs increase (34, 109, 110, 113). Accordingly, greater cannabinoid psychoactive effect is associated with greater risk for developing psychosis (10, 18, 19, 31, 33, 35, 54, 107), and individuals with schizotypal personality are more sensitive to the psychoactive effect of cannabinoids (111, 114–116). Moreover, several studies have shown that some cognitive and emotional deficits observed in chronic cannabis users are associated with schizotypal symptoms, suggesting that greater schizotypy may reflect a risk factor for the long-term adverse effects of cannabinoids (111, 115, 116). It is reasonable to assume that the psychoactive features of SC drugs along with the schizotypy characteristics of SC users, which were demonstrated in the current study, may underlie the severe adverse effects that have been associated with SCs, especially the high rates of prolonged psychotic episodes. The relationship between cannabinoid use and psychosis is well documented. Yet, the nature of the relationships between cannabinoid consumption and psychotic proneness is not fully understood (34, 86, 117). However, the phenomenon could be explained by three possible mechanisms: a) direct pharmacological effects of cannabinoids lead to schizotypal traits; b) schizotypal traits lead to cannabinoids use; or c) further factors influences both tendency toward psychosis or schizotypal traits and cannabinoids use (27). Recent data from clinical and pre-clinical studies show that acute consumption of cannabinoid agonists tend to induce brief psychotic symptoms in both vulnerable and healthy individuals (112, 117). Accordingly, when absorbed, cannabinoid agonists stimulate brain’s CB_1_ receptors which in turn modulate the firing rates of dopaminergic neurons in the ventral tegmental area—mesolimbic circuitry (34, 35). This pharmaco-dynamic mechanism may explain the short-term psychotic-like effects induced following cannabinoid-based drugs, yet there is a limited evidence for this relation in term of a long-term effect (33, 35). An alternative explanation is that individuals with schizotypal traits may use cannabinoids in order to “self-medicate” their schizotypal symptoms. Accordingly, individuals with schizotypal personality could attempt to reduce their negative symptoms by consuming cannabinoids, in order to return their control over their mental distress (27). Interestingly, earlier studies have indicated that SC users reported that despite the adverse effects, SC drugs induce pleasurable experiences such as: good mood, relaxation, and clear thought (12, 15, 88). Accordingly, beside drug-related prosecution issues, SC users most commonly consume these drugs in order to obtain positive effects, although the acute effect of SCs is unpredictable (6, 11, 14, 15, 50, 59). These results, together with the high scores of neuroticisms may support the view that SC users may use SC in an attempt to acquire a mental relief and to reduce their mental distress.

### The Relationship Between Personality Factors and Schizotypy

For both natural cannabis users and SC users, openness to experiences, and lower conscientiousness predicted schizotypy. This observation may indicate a partial common mechanism that underlies schizotypy features in these two groups. The correlation between openness to experience and schizotypy in the general population was recognized in previous studies and reflect idiosyncratic cognitive processes, unconventional ideas and elevated risk for developing schizophrenia (118, 119). Low levels of conscientiousness were also associated previously with schizotypal symptoms in non-clinical and clinical populations (119). A positive correlation between neuroticism and schizotypy for natural cannabis users is unsurprising given the association between negative affect and greater risk for developing psychosis (25). Yet, it is possible that this relationship was not observed for SC users due to their elevated levels of anxiety and depression that reduce the effect of neuroticism on schizotypy in the present study.

Notably, natural cannabis users did not differ from non-users in schizotypal measures. Although elevated schizotypal measures were previously observed among chronic cannabis users, recent studies have shown inconsistent findings. In few studies there were lower scores of negative symptoms of schizotypy in natural cannabis users compared to healthy control subjects (120, 121). Yet, earlier observations have indicated greater scores on either positive or negative schizotypal symptoms in natural cannabis users (25, 65, 108, 110). An alternative explanation to this inconsistency is that in most of these studies, additional factors which are associated with schizotypal traits such as: alcohol consumption, depression, and anxiety symptoms or current use of additional substances were not recorded or controlled (25). Finally, recent studies showed no differences in schizotypy measures between natural cannabis users and healthy control participants (122), an observation that may suggest the involvement of other moderators in this association. The presented result support this view, since after controlling for anxiety and depression levels, different traits predicted schizotypy for natural cannabis users and control participants, indicating further evidence for the complex relationships between these factors.

### Limitations of the Current Study

Studying drugs use, which is a prohibited behavior, by self-reported measures may be biased by subjective factors such as: social desirability, poor insight, and impression management (123). However, there is a consensus that self-report methods for assessing substance users have validity and reliability similar to that of biomarkers of drug consumption (63). Moreover, although the anonymity of the participants was kept in the current study, which may help to reduce socially desirable responses, we were unable to control over subjective biases, and objective measures of cannabinoids use as well as the possible use of additional psychoactive compounds. Future studies may use additional measures, such as biological assays of drugs in order to improve the reliability of the data. Secondly, the association between SCs and BFI factors was diminished when anxiety or depression were entered to the model as covariates, this result is not surprising as there is a large agreement that psychological distress, anxiety, and depression are correlated with personality dimensions such as; low conscientiousness, neuroticism, and introversion, yet, there is still a debate regarding the nature of this association in terms of causality and the involvement of additional factors in this phenomena (124). Thirdly, the current study showed an association between schizotypy and SC use, but it does not provide evidence for the direction of the relationship as the data are correlational, and therefore it is impossible to conclude whether prolonged SC use leads to schizotypy or the opposite. Future studies may consider conducting longitudinal study designs in order to better address these issues. Finally, the sample size of the current study is not large and the cross-sectional design does not allow for causal inferences. Chronic SC users are a very unique and rare cohort and difficult to recruit, therefore unfortunately our sample size was limited. Future studies may consider replicate our study using larger samples in order to confirm or disprove the current results.

## Conclusions

In conclusion, the current study provides initial evidence for the association between specific personality characteristics, schizotypal traits, and chronic SC use. On the BFI, SC users showed higher scores of neuroticism than natural cannabis users and non-users. SC users had lower agreeableness and introversion scores than both control groups, while natural cannabis users had higher extroversion scores than non-users. In addition, SC users had lower scores on conscientiousness than non-users. These effects were diminished when anxiety and depression scores were used as covariates. On the SPQ-B, SC users presented more schizotypal symptoms than both control groups. Finally, there were no differences between non-users and natural cannabis users in other personality variables. In addition, elevation of depressive and anxiety levels was observed in SC users. For SC and natural cannabis users, high measures of openness and low measures of conscientiousness have predicted schizotypy. To the best of our knowledge this is the first study presenting the complex relationships between specific personality characteristics, schizotypal traits, and SC use. The present results add initial information of the personality factors associated with SC use and their association with psychosis proneness. Yet, further studies are needed to replicate and expand the current observations.

## Data Availability Statement

The datasets generated for this study are available on request to the corresponding author.

## Ethics Statement

The studies involving human participants were reviewed and approved by the Ministry of Health, Jerusalem, Israel. The patients/participants provided their written informed consent to participate in this study.

## Author Contributions

All the authors contributed substantially to the conception and design of the study. KC and SR have collected the data. KC and AvW were responsible for the analysis of the results. All the authors contributed to further drafts of the manuscript.

## Conflict of Interest

The authors declare that the research was conducted in the absence of any commercial or financial relationships that could be construed as a potential conflict of interest.
